# Large genomic differences between *Moraxella bovoculi* isolates acquired from the eyes of cattle with infectious bovine keratoconjunctivitis versus the deep nasopharynx of asymptomatic cattle

**DOI:** 10.1186/s13567-016-0316-2

**Published:** 2016-02-13

**Authors:** Aaron M. Dickey, John D. Loy, James L. Bono, Timothy P. L. Smith, Mike D. Apley, Brian V. Lubbers, Keith D. DeDonder, Sarah F. Capik, Robert L. Larson, Brad J. White, Jochen Blom, Carol G. Chitko-McKown, Michael L. Clawson

**Affiliations:** United States Department of Agriculture (USDA), Agricultural Research Service (ARS), U.S. Meat Animal Research Center (USMARC), State Spur 18D, Clay Center, NE 68933 USA; School of Veterinary Medicine and Biomedical Sciences, University of Nebraska-Lincoln, Lincoln, NE 68583-0907 USA; College of Veterinary Medicine, Kansas State University, Manhattan, KS 66506 USA; Kansas State Diagnostic Laboratory, Kansas State University, Manhattan, KS 66506 USA; Bioinformatics and Systems Biology, Justus-Liebig-University Giessen, 35392 Giessen, Germany

## Abstract

*Moraxella bovoculi* is a recently described bacterium that is associated with infectious bovine keratoconjunctivitis (IBK) or “pinkeye” in cattle. In this study, closed circularized genomes were generated for seven *M. bovoculi* isolates: three that originated from the eyes of clinical IBK bovine cases and four from the deep nasopharynx of asymptomatic cattle. Isolates that originated from the eyes of IBK cases profoundly differed from those that originated from the nasopharynx of asymptomatic cattle in genome structure, gene content and polymorphism diversity and consequently placed into two distinct phylogenetic groups. These results suggest that there are genetically distinct strains of *M. bovoculi* that may not associate with IBK.

**Electronic supplementary material:**

The online version of this article (doi:10.1186/s13567-016-0316-2) contains supplementary material, which is available to authorized users.

## Introduction

Infectious bovine keratoconjunctivitis (IBK), commonly known as “pinkeye” in cattle, is an economically significant disease which causes pain [1], may lead to blindness and is an animal welfare concern. *Moraxella bovoculi* is a recently described bacterial species that associates with outbreaks of IBK [2]. This new species of *Moraxella* can be distinguished from two other *Moraxella species*, *M. bovis* and *M. ovis*, on the basis of phenylalanine deaminase activity, as well as divergence at 6 housekeeping genes, and genetic variation within a large ribosomal RNA (rRNA) encoding locus [3].

*Moraxella bovoculi* has not been reported to cause IBK. The type strain (ATCC BAA-1259), which was isolated from an IBK case [3], did not cause IBK in an experimental challenge study [1]. However, *M. bovoculi* isolates do contain known pathogenesis factors including a Repeats-in-Toxin (RTX) class operon which encodes a cytotoxin that lyses and kills neutrophils and corneal epithelial cells [4, 5], and a pilin (fimbriae) gene which is required for adherence to the corneal epithelium by *M. bovis* [6, 7]. *M. bovis*, is the only *Moraxella* species known to cause IBK [8], however, it has only been isolated from a small fraction of IBK case ocular secretions (reviewed in [3]). In contrast *M. bovoculi* has been frequently isolated from IBK case ocular secretions [9].

The extent of host range, niche specialization, and genetic diversity of *M. bovoculi* is unknown. In addition to IBK cases, *M. bovoculi* has been detected in ocular secretions from horse and reindeer conjunctivitis cases [10, 11], IBK asymptomatic cattle [12], as well as human respiratory tracts [13] and dog teeth [14]. Given that *M. bovis* has been found inhabiting the nasal passages of calves asymptomatic of IBK [15] this environment could be a reservoir for *M. bovoculi* as well. Taken together, the diversity of hosts and regions colonized by *M. bovoculi*, as well as the presence of known virulence factors in some isolates, indicate that there could be distinct strains of *M. bovoculi* that vary by their propensity to associate with IBK.

In this study, finished (sequenced, circularized and annotated) genomes of seven *M. bovoculi* isolates were produced and compared to each other by gene content, genome structure, single nucleotide polymorphism (SNP) diversity and evolutionary relatedness. Three of the isolates originated from the eyes of cattle afflicted with IBK and four from the nasopharynx of cattle that were asymptomatic for IBK (Additional file 1). Six of the seven isolates were spatially and epidemiologically unlinked to each other, facilitating a robust comparison between the isolates without geography or outbreak as confounding factors. Profound differences in gene content, synteny and SNP diversity were found between isolates that originated from the eyes of cattle with IBK versus those that originated from the nasopharynx of asymptomatic cattle. These results indicate that different strain types of *M. bovoculi* may vary by their frequencies within the nasopharynx and the eyes of cattle, and that some types may not associate with IBK.

## Materials and methods

### Isolate identification and selection for sequencing

The seven isolates sequenced in this study are referred to as either “IBK eye isolates” or “IBK asymptomatic nasopharyngeal isolates” throughout the manuscript. The three sequenced IBK eye isolates were obtained from ocular swabs taken from cattle afflicted with IBK in Virginia, Nebraska and Kansas. The swabs were sent to the University of Nebraska-Lincoln Veterinary Diagnostic Center by submitting veterinarians for diagnostic assessment of IBK outbreaks. The four sequenced IBK asymptomatic nasopharyngeal isolates were obtained from deep nasopharyngeal swabs of cattle that were asymptomatic for IBK. Three of the IBK asymptomatic nasopharyngeal isolates were sampled from cattle located at Missouri, Kentucky and Tennessee sale barns prior to transport to a facility in Kansas. A fourth isolate was obtained from a calf that originated in the same Missouri sale barn but was sampled upon arrival at the Kansas facility. Use of these cattle for nasopharyngeal sampling and microbial identification was approved by the Institutional Animal Care and Use Committee of Kansas State University.

All isolates were identified as *M. bovoculi* based on colony morphology on tryptic soy agar plates with 5% sheep blood, oxidase production, a negative gram stain, cell morphology (Additional file 1), matrix assisted laser desorption ionization time-of-flight mass spectrometry (MALDI-TOF MS) and by species-level identification in at least one of two diagnostic polymerase chain reaction (PCR) assays. The MALDI-TOF MS assay was used for genus (*Moraxella*) level identification only. For the assay, individual colonies were selected and transferred to a 96 spot stainless steel target. A 1 µL overlay of α-cyano-4-hydroxycinnamic acid (HCCA) matrix (Bruker Daltonik, Billerica, MA, USA) was placed on each bacterial sample. Instrument calibration was performed using standard reference BTS *Escherichia coli* (Bruker). Mass spectra were acquired using a MALDI-TOF MF, Microflex LT system in a linear positive mode (Bruker). Spectra were matched to MALDI Biotyper 3.0, Reference Library 1.0 Version 3.1.2 for identification. A cut-off score >2.000 was considered a secure genus level identification. Of the two PCRs that tested for *Moraxella* species identification, one incorporated a restriction fragment length polymorphism (RFLP) analysis performed on a PCR amplified locus containing the 16S rRNA gene, partial 23S rRNA gene and the intergenic spacer [9, 16]. The second was an in-house diagnostic PCR that was developed at the Kansas State Diagnostic Laboratory (Chengappa et al. unpublished).

### DNA purification and sequencing

To obtain DNA for sequencing, all seven *M. bovoculi* isolates were grown from frozen culture stocks on chocolate agar plates at 37 °C with 5% CO_2_ for 17–20 h. A single colony for each isolate was then picked and further passaged a minimum of one additional time on chocolate agar. The twice-purified colonies were subsequently transferred to brain heart infusion (BHI) broth and harvested at mid-log growth phase as determined using a GENESYS 20 spectrophotometer (Thermo Fisher, Waltham, MA, USA). The cultured cells were collected by centrifugation and their DNA was extracted via G-100 columns (Qiagen, Valencia, CA, USA) as previously described, with a slight modification [17]. Approximately 10 µg of DNA for each isolate was sheared to ~20 kbp in a g-tube (Covaris, Woburn, MA, USA). Single molecule real-time DNA libraries (SMRT Bell 1.0, 10–20 kbp insert size) were prepared from the sheared DNA according to the manufacturer’s instructions (Pacific Biosystems, Menio Park, CA, USA) and sequenced with a 4 h movie time on a PacBio RSII DNA sequencer. An older P5/C3 PacBio chemistry was used for IBK asymptomatic nasopharyngeal isolate Mb33362 (sequenced October, 2014) and a newer P6/C4 chemistry was used for the remainder of the isolates (sequenced April, 2015). Both chemistries resulted in DNA read lengths that supported whole genome assemblies of the isolates. In addition to RSII sequencing, Nextera XT libraries were generated and sequenced on a MiSeq instrument (Illumina, San Diego, CA, USA) for 5 of the isolates for error correction of the corresponding PacBio assembled genome sequence.

### Genome assembly and annotation

To assemble, close and annotate the genomes for all seven isolates, the largest reads were error-corrected and assembled into a single contig using the RS_HGAP_Assembly.3 protocol from SMRT Analysis Software v2.3.0 then validated and improved with Quiver [18]. Default parameters of the protocol were used except for the predicted genome size, which was set to 2.3 Mbp. This value was increased to 5 Mbp when the contigs produced did not have overlapping ends in order to incorporate additional reads into the assembly. The preliminary contigs ranged in size from 2.14 to 2.42 Mbp and had overlapping ends ranging from 6.9 to 17.4 kbp with self–self identity of 99.5–99.8%. This indicated that the contigs represented completely sequenced closed genomes in need of overlapping-end-trimming. In addition, a single plasmid was identified from IBK eye isolate Mb58069 (Additional file 2). The preliminary contig for this plasmid was 69.7 kbp and had a 19.4 kbp terminal overlap with 99.6% self–self identity. Error-corrected read coverage ranged from 10-42X and N50 read lengths ranged from 13.1 to 27.8 kbp. All preliminary contigs with overlapping ends were initially annotated with the software “Do It Yourself Annotator” (DIYA) [19]. The Ori-Finder [20] web server was used to identify the putative *oriC* region. From three putative *oriC* regions identified in isolate Mb22581, a region immediately preceding the Chromosomal replication initiator protein *dnaA* coding sequence (CDS), which was identified by DIYA, was selected as the origin for isolate Mb22581 and the other six *M. bovoculi* genomes. The origin of replication of the plasmid was set at position 1 of the Initiator protein *repB* CDS. Contig trimming, setting the origin and self–self dot plot analyses were conducted in Geneious v8 (Biomatters, Auckland, New Zealand). To correct sequencing errors and produce higher overall fidelity whole-genome sequences, the trimmed contigs were used as reference sequences for re-mapping all of the PacBio reads (RS_Resequencing.1 protocol+Quiver) to produce finished assemblies. The finished assemblies were checked with corresponding mapped MiSeq reads for any additional error correction in Geneious. Unannotated contig sequences were deposited in GenBank and annotated according to the NCBI prokaryotic genome annotation pipeline [21]. The size and accession number(s) of each isolate genome are given in Additional file 2. The genomes were also annotated with the RAST web server [22]. CDS counts from the three annotations (DIYA, GenBank and RAST) are provided in Additional file 2.

### Phylogenetic analyses

Two separate phylogenetic analyses were performed on all seven *M. bovoculi* isolates sequenced in this study along with additional DNA sequences from *M. bovoculi*, *M. ovis* [3], *M. bovis* (3, Dickey et al. unpublished), *M. caprae* [23] and *M. boevrei* [23]. The first analysis utilized the same ribosomal DNA (rDNA) locus employed in the PCR–RFLP test [16] and in the initial description of *M. bovoculi* [3] (16S, partial 23S and the intergenic spacer). The second analysis compared the rDNA tree with gene trees from four housekeeping genes used in the initial description of *M. bovoculi* [3]. Two additional housekeeping genes [3] were absent or had suspect homology in the *M. bovis* genome (Dickey et al. unpublished) and were therefore not used. Single orthologous genes were identified in each genome using the map-to-reference tool in Geneious and were also identified in the draft genomes of *M. boevrei* and *M. caprae* [23] using BLAST [24]. All *M. bovis* genes were 100% sequence identity matches to homologous gene sequences of *M. bovis* isolate Tifton I [3]. Multiple sequence alignments were conducted with MUSCLE [25]. The rDNA locus was analyzed as two partitions with the gaps extracted from the alignment (multi-locus gap alleles setting in DnaSP v5 [26]) and treated as a separate binary data partition. The best model of sequence evolution for the rDNA was tested with jModelTest [27]. The housekeeping genes were individually partitioned according to RAXML criteria for best sequence evolution model and partition scheme in PartitionFinder [28] with each codon position and non-coding region treated as potential partitions.

Best fit models and partitions were used in RAxML v8 [29] analyses to generate maximum-likelihood trees for each locus with 1000 bootstrap pseudoreplicates and SH-like approximate likelihood ratio test (aLRT) [30] for nodal support. Nodes where either support value was <0.50 were collapsed in Mesquite v3.04 [31] and final trees were visualized in FigTree v1.4 [32]. To compare the five gene trees (rDNA plus four housekeeping genes), SplitsTree v4 [33] was used to generate a super-network of all compatible splits [34]. The network was then collapsed down to a simpler tree by (a) keeping compatible splits in decreasing order of weight and (b) calculating a consensus tree.

### Identification of the *M. bovoculi* core and pan-genomes, and predicted biochemical pathways

Using GenBank annotated genomes, the “Efficient Database framework for comparative Genome Analyses using BLAST score Ratios” or “EDGAR” web interface [35] was used to calculate the *M. bovoculi* pan-genome as well as core genomes corresponding to IBK eye isolates, IBK asymptomatic nasopharyngeal isolates, and all *M. bovoculi* isolates. The available draft genome sequence of the type strain (ATCC BAA-1259) [6] was included in EDGAR calculations, with its existing annotation, along with the sequences generated in this study. Singleton genes were defined as genes without any BLASTP hit exceeding the calculated orthology threshold in any other genome and the singleton decay function was calculated because of its contribution to the (theoretically infinite) open pan-genome size of bacterial species. The Heaps’ law function of the *M. bovoculi* open pan-genome as a function of genome number and decay functions for singletons and the core *M. bovoculi* genome was subsequently calculated. EDGAR and Geneious were also used to identify the non-homologous IBK eye isolate core genome (bi-directional best BLASTP hit genes in all IBK eye isolate genomes with no BLASTP hits to any IBK asymptomatic nasopharyngeal isolate genomes) and the non-homologous IBK asymptomatic nasopharyngeal isolate core genome as subsets of the IBK eye isolate core and IBK asymptomatic nasopharyngeal isolate core genomes.

The genes comprising the non-homologous IBK asymptomatic nasopharyngeal isolate core genome and the genes comprising the non-homologous IBK eye isolate core genome were submitted to pathway analysis to predict possible biochemical functions encoded by these genes. RAST annotations were used in pathway analysis as these contained more functional labels than the other two annotations. Pathway analysis consisted of generating level 3 Pathway/Genome databases with PathoLogic in Pathway Tools 19.0 [36] using MetaCyc 19.0 [37].

### Identification of genome blocks and rearrangement patterns between *M. bovoculi* isolates

In order to identify and compare patterns of genome rearrangement caused by homologous recombination within and between the isolates, localized collinear blocks of the seven genomes were identified using the Mauve [38] plug-in of Geneious. The draft genome (ATCC BAA-1259) was not included in this analysis as it has not been circularized. To investigate possible correlations between genome rearrangements and prophages, phage elements were identified using the PHAST web server [39].

Based on the observation of a single generalized synteny in IBK eye isolate *M. bovoculi* isolate genomes, a single circularized contig was created from the contigs of the published *M. bovoculi* type strain draft genome, (ATCC BAA-1259, GenBank# AOMT00000000) [6]. This contig contained all but singleton genes (contigs 6, 17, 38, 41 and 42). A maximum-likelihood phylogenomic tree was constructed with the FastTree 2 [40] plug-in of Geneious from SNPs that were extracted from a Mauve alignment of all eight genomes with SH-like aLRT nodal support.

### Detection of known pathogenesis factors

The presence or absence and associated structure of previously identified pathogenesis factors, the RTX operon and pilin gene, was determined among isolates. To facilitate this, genome alignments were conducted with MUSCLE and Clustal [41] and refined manually for detection and quantification of these differences. These calculations did not include the type strain, ATCC-BAA-1259, since the RTX operon and pilin gene were already known to be present in this isolate [4, 6].

### Detection of antibiotic resistance genes and in vitro antimicrobial sensitivity assays

ResFinder [42] was used to identify acquired antibiotic resistance genes, which indicated the presence of an antibiotic resistance gene cluster within the IBK eye isolate Mb58069 genome and prompted characterization of all isolates via antibiotic susceptibility assays. To characterize in vitro antimicrobial sensitivity, a broth microdilution system was utilized following Clinical Laboratory Standards Institute (CLSI) guidelines (VET01-A4). Several colonies of pure culture were suspended into 10 mL of sterile demineralized water to a 0.5 McFarland standard and vortexed to ensure uniform resuspension. Inoculation density was confirmed using a calibrated nephelometer. A 10-µL aliquot of the resuspended organisms was then inoculated into 11 mL of sterile inoculation media and vortexed to ensure uniform resuspension. A 100-µL aliquot of culture per well was then inoculated into bovine and/or porcine antimicrobial susceptibility panels (Trek Diagnostics, Thermo Fisher, Waltham, MA, USA) with an autoinoculator. The samples were incubated at 35 °C for 18 h without carbon dioxide supplementation and were read automatically with the minimal inhibitory concentration (MIC) determined using an automated system (Sensititre ARIS 2X, Thermo Fisher, Waltham, MA, USA). The auto-read values were confirmed manually by observation as necessary. Quality control organisms utilized for the assays included *Staphylococcus aureus* (ATCC 29213), *Enterococcus faecalis* (ATCC 29212) and *Escherichia coli* (ATCC 25922). No specific CLSI-approved interpretative criteria existed for *Moraxella* spp. in cattle at the time this work was performed, therefore interpretive criteria established for bovine respiratory disease or other Gram-negative veterinary isolates were used as available [43] (Additional file 3), with organisms classified as susceptible (S), intermediate (I), or resistant (R). Breakpoints were not available for neomycin and tylosin. For trimethoprim–sulfamethoxazole and sulfadimethoxine only a single drug concentration was tested.

The plasmid of IBK eye isolate Mb58069 was characterized by performing a BLASTN [24] search against the nr database.

## Results

### Generation of circularized genomes and results of diagnostic PCRs and phylogenetic analyses for the seven *Moraxella bovoculi* isolates

**Figure 1:**
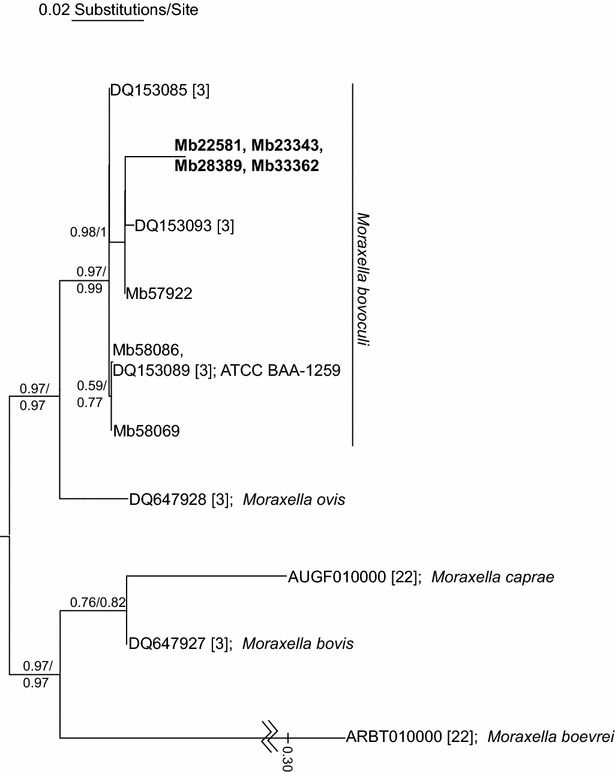
**Maximum-likelihood tree of the large (3430–3895** **bp) ribosomal DNA locus previously used to describe** ***Moraxella bovoculi***. Branch lengths are shown to-scale with the exception of *M. boevrei*. Taxon labels are either GenBank accession numbers from [3, 22] or isolate numbers from the present study; all three *M. bovoculi* from [3] are ocular isolates from clinical samples. The four IBK asymptomatic nasopharyngeal *M. bovoculi* isolates are in bold. Support values subtending nodes are non-parametric bootstrap proportions followed by SH-like aLRT support. Nodes with support values below 0.5 have been collapsed.

High quality circularized genomes were obtained from all seven isolates of *M. bovoculi* sequenced in this study. Fold-coverage across the seven genomes and one plasmid ranged from 80 to 372 (median 289) and the accuracy ranged from 99.9880 to 99.9999% (median 99.9994%). The Kansas State Diagnostic Laboratory PCR identified all isolates as *M. bovoculi* and the PCR–RFLP identified all of the IBK eye isolates as *M. bovoculi*. The IBK asymptomatic nasopharyngeal isolates produced a single PCR–RFLP band ranging from 657 to 663 nucleotides in length that was identifiable as *Moraxella*, but inconclusive as to the species. A maximum-likelihood tree of the same rDNA locus used for the initial description of *M. bovoculi* as a new species [3] was constructed for the seven *M. bovoculi* sequenced in this study as well as sequences from other *M. bovoculi* isolates used in the initial description and other *Moraxella* species. The rDNA tree resolved the IBK asymptomatic nasopharyngeal isolates within a monophyletic clade that also contained all of the IBK eye isolates and previously characterized *M. bovoculi* isolates with strong bootstrap and SH support (Figure 1). Four housekeeping genes that have also been previously used to describe *M. bovoculi* yielded conflicting phylogenetic signals regarding placement of the IBK asymptomatic nasopharyngeal isolates in relation to the IBK eye isolates on a phylogenetic network. The IBK asymptomatic nasopharyngeal isolates either placed within a monophyletic clade with other *M. bovoculi* isolates, or in a basal position to other *M. bovoculi* (Additional file 4). Thus, phylogenetic signal between the housekeeping genes of the isolates sequenced in this study was somewhat ambiguous. In contrast, the rDNA locus, which is commonly used for prokaryotic identification and taxonomic placement, unequivocally supported the identification of all seven isolates sequenced in this study as *M. bovoculi*.

### Identification of the *Morxella bovoculi* pan and core genomes, and genome structure

**Figure 2:**
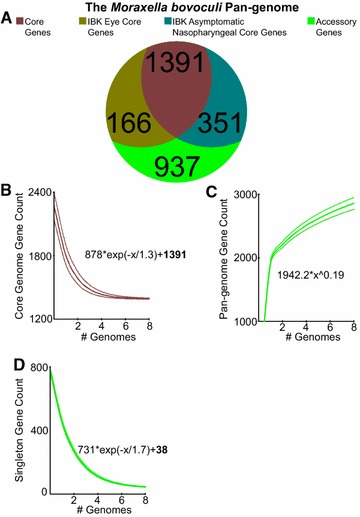
**The** ***Moraxella bovoculi*** **Pan-genome**. **A** The pan-genome is subdivided and color Coded according to the core genome, the IBK eye and IBK asymptomatic nasopharyngeal core genomes and the accessory genome. The accessory genome contains all non-core genes. Development plots are shown for the *M. bovoculi* **B** core genome, **C** pan-genome and **D** singleton genes with 95% confidence intervals as dotted lines and where x is equal to the number of genomes. Asymptotic regression coefficients in their respective equations are in bold.

**Figure 3:**
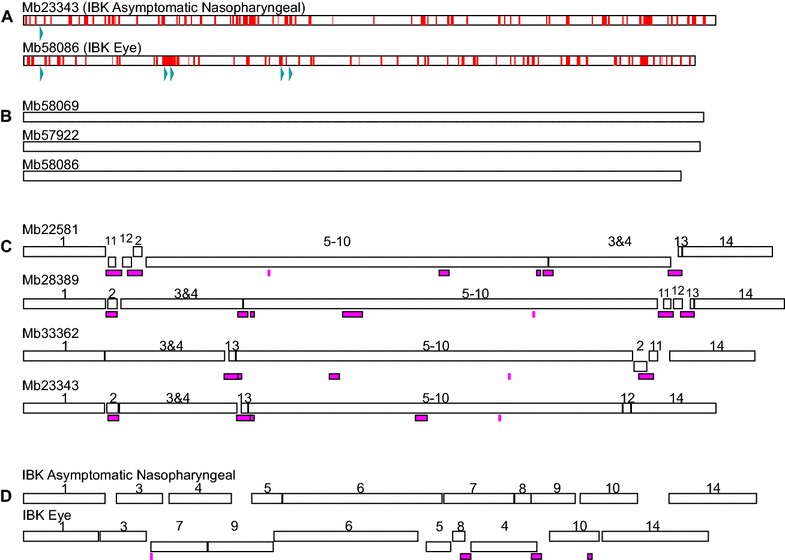
**Genome-scale structure patterns within and among three IBE eye** ***M. bovoculi*** **isolates and four IBK asymptomatic nasopharyngeal** ***M. bovoculi*** **isolates**. **A** Gene distribution of the non-homologous IBK eye (*n* = 129) and IBK asymptomatic nasopharyngeal (*n* = 189) core genes within representative genomes. CRISPR regions are indicated with blue arrows. **B** Absence of genome rearrangement among individual clinical genomes. **C** Four >20 kbp blocks (2, 11–13) of the non-clinical genome are translocated and/or inverted (block appears beneath neighboring blocks) among Mb28389, Mb23343 and Mb33362 genomes whereas Mb22851 exhibits a single large inversion of blocks 3–10. Several breakpoints correlate with 25 phage elements (pink). **D** Four >25 kbp blocks (4, 5, 7 and 9) are translocated and inverted between the consensus IBK eye genome and the consensus IBK asymptomatic nasopharyngeal genome (IBK asymptomatic nasopharyngeal blocks 2, 11–13 not shown). Several breakpoints correlate with four phage elements (pink) identified within Mb57922 and Mb58069 clinical genomes.

In the pan-genome analyses, *M. bovoculi* isolates from IBK eye isolates were found to differ in gene content from the IBK asymptomatic nasopharyngeal isolates. Overall, the *M. bovoculi* core genome contained 1391 genes (Figure 2A) and was not predicted to regress further with the addition of more genomes (Figure 2B). However, the four IBK asymptomatic nasopharyngeal isolate genomes had an additional core of 351 genes, of which 189 lacked a homolog in the IBK eye isolates. The four IBK eye isolate genomes (three sequenced here plus ATCC BAA-1259) also had an additional core of 166 genes, of which 129 lacked a homolog in IBK asymptomatic nasopharyngeal isolates. Two clusters of non-homologous IBK eye isolate core genes associated with clustered regularly interspaced short palindromic repeats (CRISPR) elements (Figure 3A), which had a fivefold greater abundance in IBK eye isolate genomes (Additional file 2). In summary, 8.29% of the genes comprising the *M. bovoculi* IBK eye isolate core genome had no homolog in *M. bovoculi* IBK asymptomatic nasopharyngeal isolates, and 10.85% of the genes comprising the core genome of *M. bovoculi* IBK asymptomatic nasopharyngeal isolates had no homolog in IBK eye isolates.

In the pathway analysis, The 189 genes comprising the non-homologous IBK asymptomatic nasopharyngeal core genome and the 129 genes comprising the non-homologous IBK eye core genome were computationally predicted to encode divergent sets of biochemical functions (Additional file 5). Among the pathways predicted in the IBK eye core genome are those for heavy metal detoxification and antibiotic resistance.

In the genome rearrangement analysis, a total of 29 localized collinear blocks were identified in all genomes. Of these, 14 had consistent homology for >20 kbp and a summary of genome rearrangements among these blocks is presented in Figure 3. Briefly, the genomes of *M. bovoculi* IBK eye isolates appeared constrained to a single generalized synteny whereas the genomes of *M. bovoculi* IBK asymptomatic nasopharyngeal isolates were highly variable with regard to genome rearrangements. Furthermore, rearrangement of four large (70–220 kbp) localized collinear blocks demarcated the divergence between the genomes of IBK eye and IBK asymptomatic nasopharyngeal isolates from a common ancestor, and the breakpoints for genome rearrangements were physically correlated with phage elements (Figures 3C and D). There were, on average, more than four times as many phage elements identified within the genomes of IBK asymptomatic nasopharyngeal isolates than there were in the genomes of IBK eye isolates (Additional file 2).

### Phylogeny of isolates using genome-wide SNPs

**Figure 4:**
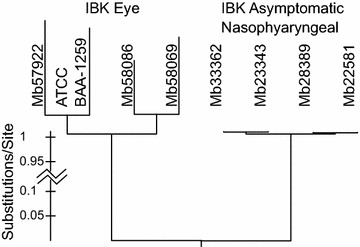
**Mid-point rooted maximum-likelihood phylogenetic tree of 81** **284** ***Moraxella bovoculi*** **SNPs from eight genomes**. The tree shows a 12–239 fold increased rate of nucleotide substitution in the IBK eye clade compared to the IBK asymptomatic nasopharyngeal clade. aLRT support for all splits = 100.

A maximum-likelihood phylogenomic tree was constructed from 81 284 SNPs extracted from eight genomes (seven sequenced here plus the draft *M. bovoculi* type strain genome). Two striking patterns emerged from the tree (Figure 4): (1) the majority of SNPs separated the genomes of IBK eye isolates and those of IBK asymptomatic nasopharyngeal isolates into clades and (2) the rate of nucleotide substitution was 1–2 orders of magnitude higher within the IBK eye isolate clade. All nodes within the tree were fully supported indicating strong phylogenetic signal and no phylogenetic conflict.

### Detection of antibiotic resistance genes, other virulence factors and plasmids

The *M. bovoculi* genomes differed by the presence or absence of the RTX operon, a known pathogenesis factor The RTX operon was absent from the genomes of all isolates obtained from the nasopharynx of IBK asymptomatic animals. Of the three IBK eye isolates, Mb58069 and MB57922 had complete RTX operons, whereas Mb58086 did not contain the RTX operon within its genome. This indicates that the RTX operon is not absolutely required by *M. bovoculi* isolates that occupy the eyes of cattle afflicted with IBK. The pilin A gene, another known virulence factor, was present in all genomes but IBK eye isolates differed from IBK asymptomatic nasopharyngeal isolates at 144 of 459 nucleic acid sites (31.3%) at that locus, and their corresponding predicted protein isoforms differed by 36 of 152 amino acids (23.6%).

ResFinder was used to identify acquired antibiotic resistance genes and these were found only in IBK eye isolate Mb58069. A total of 10 co-located antibiotic resistance genes (Additional file 6) were identified in an ~27 kbp genomic island of the Mb58069 genome. All isolates showed an intermediate MIC response to clindamycin, however, Mb58069 was resistant to florfenicol, oxytetracycline, sulfadimethoxine and trimethoprim-sulfamethoxazole (Additional file 7). In addition, this isolate had an elevated MIC value in the intermediate category to the macrolides tilmicosin and tulathromycin (Additional file 7).

The plasmid of isolate Mb58069 was characterized with a BLASTN search. The most similar plasmid in GenBank was from *Pseudomonas aeruginosa*. The highest scoring 20 plasmids in this search represent 6 genera of Gammaproteobacteria with E-values ranging from 1^−102^ to 2^−30^ and are summarized in Additional file 8.

## Discussion

In this study, multiple differences were found between *M. bovoculi* genomes of isolates obtained from the eyes of cattle with clinical IBK versus the nasopharynx of asymptomatic cattle that are both large in amplitude and striking in evolutionary pattern. More than 81 000 SNPs were identified, and over half define an evolutionary branch that bifurcates these isolates into two major clades, predicting the genomic state of the most recent common ancestor of each clade. Additionally, the IBK eye isolate clade is characterized by an apparent elevated rate of nucleotide substitution (12–239 fold increase) in comparison to the IBK asymptomatic nasopharyngeal isolate clade (Figure 4).

The IBK eye isolate core genome contains ~10.6% fewer genes than the IBK asymptomatic nasopharyngeal isolate core genome (Figure 2). This relative size difference is consistent with a pattern of selection for gene loss in pathogens, and is also consistent with a change of lifestyle, or switch of niches within the host (reviewed in [44]). A substantial portion of the non-homologous IBK eye isolate core genome may have been integrated by CRISPR mobile elements as suggested by their proximity to CRISPRs within the genome (Figure 3A). IBK asymptomatic nasopharyngeal isolates, in addition to a higher gene count in their core genome, also show a high degree of genome rearrangement not seen in their IBK eye isolate counterparts (Figure 3). Most breakpoints appear “symmetrical” with respect to the *oriC* region consistent with other studies and theory [45, 46]. Furthermore, recombination breakpoints also correlate with the presence of phage elements, which was also seen in the human pathogen *Neisseria meningitidis* [47]. Unlike *N. meningitidis* pathogenic strains only *M. bovoculi* isolates from the nasopharynx of IBK asymptomatic cattle show a high degree of genome rearrangement, which is somewhat surprising given that genome rearrangements are a common feature of “recently emerged genetically uniform pathogens” [44]. However, recombination potential may be dependent on sequence elements within the genome rather than the ecology or virulence of the isolate [48, 49]. While IBK eye isolates appear inflexible to genome rearrangements, recombination of large sections of the genome separates the IBK eye clade from the IBK asymptomatic nasopharyngeal clade and these breakpoints also correspond to phage elements (Figure 3D).

The antimicrobial resistance data (Additional file 7) provides evidence that the genomic island identified in IBK eye isolate Mb58069 (Additional file 6) is linked to a reproducible antimicrobial resistance phenotype. This island is of clinical interest since oxytetracycline and tulathromycin are the only FDA approved antimicrobials for treatment of IBK associated with *M. bovis*. The presence and possible circulation of genomic islands within *M. bovoculi* may account for the four-fold increase in MIC_90_ values found in isolates of *M. bovoculi* for oxytetracycline and select isolates recently observed in diagnostic submissions that have elevated MIC values for tulathromycin [9].

A surprising result of this study is the complete absence of the RTX operon in one of the *M. bovoculi* IBK eye isolates. This suggests a dispensable role for the putative virulence factor in eye colonization and the presence of other virulence factors in the genome should be explored. This finding also may account for the variation in hemolysis patterns observed among *M. bovoculi* isolates when cultured on blood agar [3]. The magnitude of divergence at the pilin A gene among IBK eye and IBK asymptomatic nasopharyngeal isolates is also striking. The 31.3% difference identified is approximately seven fold higher than the average sequence difference across the *M. bovoculi* core genome and suggests that disruptive selection on portions of this gene may be playing a niche-determining role within the host. Anti-pilin immunity is critical in the host response against *M. bovis*, where immunity is strain specific amongst multiple serotypes [50, 51]. While observational data has suggested that autogenous vaccine formulations that included *M. bovoculi* antigens may show benefit [52], experimentally these vaccines have not demonstrated efficacy [53, 54]. A high degree of diversity among pilin genes may account for some of these observations as heterogenetity across pilus types between circulating and vaccine strains may reduce vaccine efficacy.

While profound differences between *M. bovoculi* isolates were found in this study, it will be important to test whether IBK asymptomatic eye isolates and IBK symptomatic nasopharyngeal isolates follow similar or novel patterns of genetic differentiation and whether some strains of *M. bovoculi* actually cause IBK. Additionally, the extent of SNP allele linkage within and between clades of *M. bovoculi* should be determined using deep populations of epidemiologically and geographically unlinked isolates. This could potentially lead to the development of a DNA-based diagnostic test that uses a minimal set of tagging SNPs to distinguish members of each clade, as has been done previously for other bacteria [17, 55]. Additionally, the divergent biochemical functions predicted for the non-homologous core genomes of each clade (Additional file 5), should be tested by phenotyping. These phenotypes could then be used to develop a field test for carriers of *M. bovoculi* strains that associate with IBK. Finally, identification of *M. bovoculi* strains that have an increased propensity to associate with IBK due to their genetic determinants may facilitate the design of efficacious vaccines that only target IBK-associating strains. Strains that do not associate with IBK may provide unknown beneficial roles to their host in complex environments such as the nasopharynx.

## Additional files

10.1186/s13567-016-0316-2 **Isolate information**. Table containing the following information related to each isolate when available: Isolate identifier, IBK eye isolates or IBK asymptomatic nasopharyngeal isolates, location, specimen type, infection characteristics and microbial phenotype. The single epidemiologically linked isolate is also noted.
10.1186/s13567-016-0316-2 **Genome information for replicons**. Table containing the following information related to each replicon: replicon type, isolate identifier, replicon size, GenBank accession number and RTX operon status. Also contained in the table are the following elemental counts: phages, CRISPRs, 16s rRNA genes, tRNA genes, the number of acquired antibiotic resistance genes and CDS counts for three genome annotation methods.
10.1186/s13567-016-0316-2 **Interpretive criteria for selected antimicrobial drugs**. Table containing the following information related to MIC testing: antimicrobial, concentration(s) tested and threshold criteria for susceptible, intermediate or resistant.
10.1186/s13567-016-0316-2 **Conflict among gene trees represented by unweighted phylogenetic networks**. A) A supernetwork showing all compatible splits shown as equal angles except for the unambiguous split separating the ingroup from the outgroup. Collapsing the network by greedily keeping compatible splits in decreasing order of weight yields network B. B) The greedy compatible network characterized by IBK asymptomatic nasopharyngeal *M. bovoculi* (thick terminal branch, taxon 11) contained within IBK eye isolates and previously characterized [3] isolates as in the ribosomal DNA locus tree (Figure 1) and the 3-Hydroxyacyl-CoA dehydrogenase gene tree (not shown). In contrast, the consensus tree C) is characterized by IBK asymptomatic nasopharyngeal *M. bovoculi* basal to all other *M. bovoculi* as in the RNA polymerase subunit B, ATP synthase F1-epsilon subunit, and Phospho-N-acetylmuramoyl-pentapeptide transferase gene trees (not shown).
10.1186/s13567-016-0316-2 **Pathway analysis of 129 genes from the non-homologous IBK eye core genome and 189 genes from the non-homologous IBK asymptomatic nasopharyngeal core** ***M. bovoculi*** **genomes**. Table containing the following information related to each pathway: name, type in parentheses and class. Pathways are subdivided into those predicted to have been acquired independently, those predicted to be encoded by the IBK eye core genome and those predicted to be encoded by the IBK asymptomatic nasopharyngeal core genome.
10.1186/s13567-016-0316-2 **Acquired antibiotic resistance genes in** ***M. bovoculi*** **IBK eye isolate Mb58069**. Table containing the following information related to acquired antibiotic resistance genes in Mb58069: name of gene, % identity, matching accession number, position in Mb58069 genome and expected resistance phenotype.
10.1186/s13567-016-0316-2 **Selected minimum inhibitory concentrations (MICs) for each isolate/antimicrobial combination**. Table containing the following information related to MIC testing for each isolate: antimicrobial, MIC and applied interpretive criteria (susceptible, intermediate or resistant) in parenthesis.
10.1186/s13567-016-0316-2 **Top Mb58069 plasmid BLASTN hits**. Table containing the following information related to the Mb58069 plasmid: BLAST hit, % identity, E-value and plasmid description.
