# Supplementary material for: Large genomic differences between Moraxella bovoculi isolates acquired from the eyes of cattle with infectious bovine keratoconjunctivitis versus the deep nasopharynx of asymptomatic cattle
Source: Vet Res. 2016 Feb 13;47:31. doi: 10.1186/s13567-016-0316-2 (PMC4752781; doi:10.1186/s13567-016-0316-2)
Supplement: Supplementary file 4 — 10.1186/s13567-016-0316-2 Conflict among gene trees represented by unweighted phylogenetic networks. A) A supernetwork showing all compatible splits shown as equal angles except for the unambiguous split separating the ingroup from the outgroup. Collapsing the network by greedily keeping compatible splits in decreasing order of weight yields network B. B) The greedy compatible network characterized by IBK asymptomatic nasopharyngeal M. bovoculi (thick terminal branch, taxon 11) contained within IBK eye isolates and previously characterized [3] isolates as in the ribosomal DNA locus tree (Figure 1) and the 3-Hydroxyacyl-CoA dehydrogenase gene tree (not shown). In contrast, the consensus tree C) is characterized by IBK asymptomatic nasopharyngeal M. bovoculi basal to all other M. bovoculi as in the RNA polymerase subunit B, ATP synthase F1-epsilon subunit, and Phospho-N-acetylmuramoyl-pentapeptide transferase gene trees (not shown). [file 13567_2016_316_MOESM4_ESM.pdf]

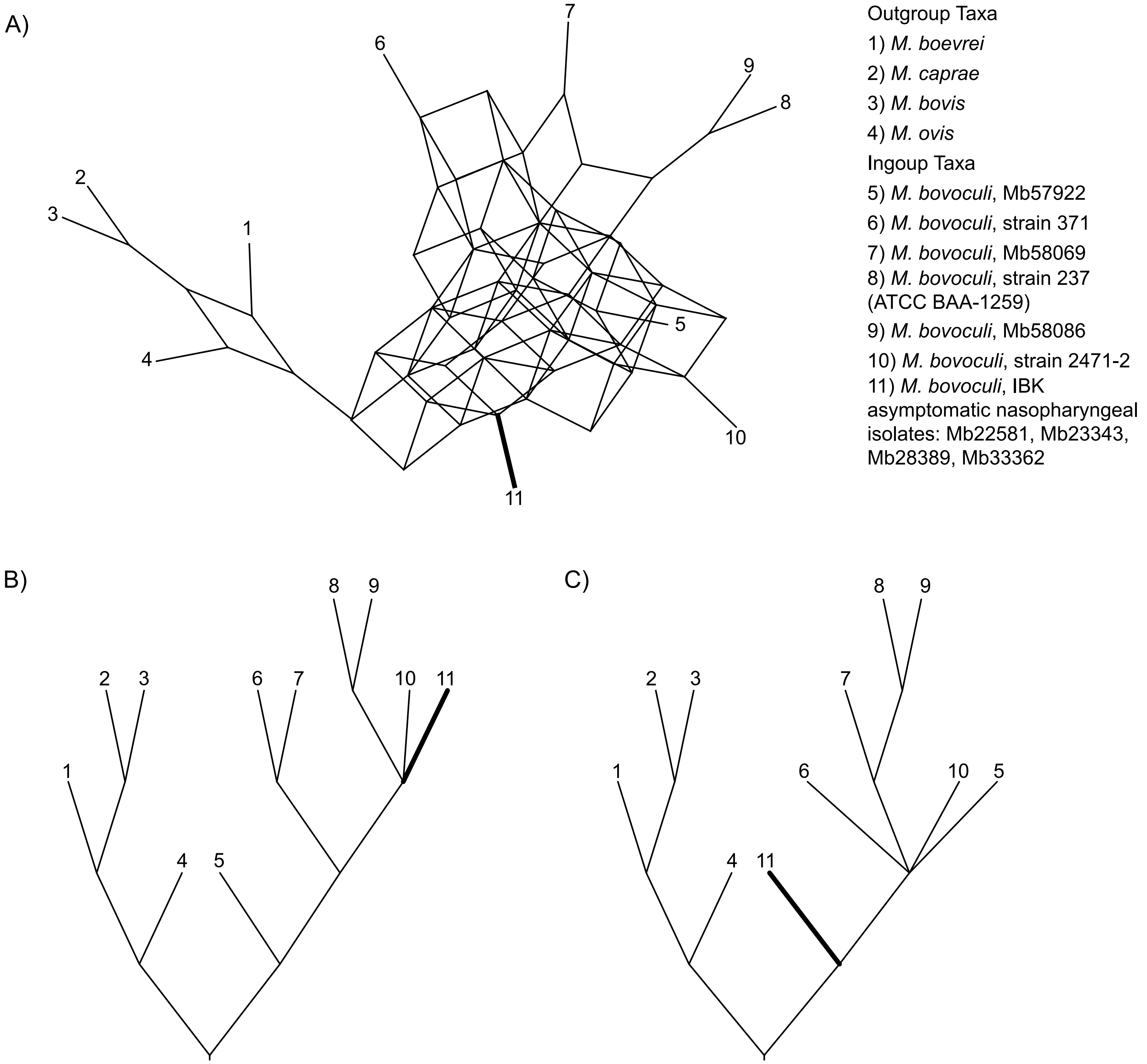
A)
B)
C)
1 2 3 4 5 6 7 8 9 10 11
Outgroup Taxa
1) M. boevrei
2) M. caprae
3) M. bovis
4) M. ovis
Ingoup Taxa
5) M. bovoculi, Mb57922
6) M. bovoculi, strain 371
7) M. bovoculi, Mb58069
8) M. bovoculi, strain 237 (ATCC BAA-1259)
9) M. bovoculi, Mb58086
10) M. bovoculi, strain 2471-2
11) M. bovoculi, IBK asymptomatic nasopharyngeal isolates: Mb22581, Mb23343, Mb28389, Mb33362
